# cuteHap: Haplotype‐Aware Structural Variant Detection in Phased Long‐Read Sequencing Data

**DOI:** 10.1002/advs.202519314

**Published:** 2026-02-12

**Authors:** Shuqi Cao, Yadong Liu, Miao Cui, Runtian Gao, Weimin Guo, Guohua Wang, Yadong Wang, Tao Jiang

**Affiliations:** ^1^ Center for Bioinformatics Faculty of Computing Harbin Institute of Technology Harbin Heilongjiang China; ^2^ Key Laboratory of Biological Bigdata Ministry of Education Harbin Institute of Technology Harbin Heilongjiang China; ^3^ Zhengzhou Research Institute Harbin Institute of Technology Zhengzhou Henan China; ^4^ College of Life Sciences Northeast Forestry University Harbin Heilongjiang China

**Keywords:** genomic analysis, haplotype‐aware, long‐read sequencing, phasing, somatic mosaicism, structural variations, variant calling

## Abstract

Long‐read sequencing has enabled comprehensive exploration of human genome at an unprecedented scale, particularly enhancing our understanding of structural variants (SVs). Phasing, a powerful approach for assigning haplotypes to sequencing reads, enables the generation of haplotype‐aware call sets without requiring whole‐genome assembly and provides a new direction for SV detection. Herein, we present cuteHap, a haplotype‐aware SV detection method designed for phased long‐read sequencing data. cuteHap fully leverages phased alignments and automatically selects a self‐adaptive clustering strategy or a cluster credibility‐prioritized beam search algorithm to achieve accurate haplotype‐resolved SV calls. In addition, cuteHap incorporates a mosaic detection module to resolve somatic mosaicism. cuteHap achieved 6% and 3% higher F1‐scores on Pacific Biosciences High‐Fidelity (PacBio HiFi) and Oxford Nanopore Technologies (ONT) datasets, respectively, and detected a greater diversity of low‐frequency SVs in tumor datasets. Its robust and high‐performance SV detection facilitates the generation of high‐quality haplotype‐resolved call sets and advancing global genomic and genetic research.

## Graphical abstract

cuteHap is a haplotype‐aware structural variant detection method designed for phased long‐read sequencing. By employing self‐adaptive clustering and credibility‐prioritized beam search algorithms, cuteHap generates accurate haplotype‐resolved calls and outperforms state‐of‐the‐art tools. Additionally, cuteHap identifies somatic mosaicism for cancer genomics. The robust performance facilitates high‐quality call sets, thereby advancing high‐quality genetic research.

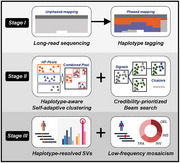

## 1 Introduction

Structural variations (SVs) are a major class of genomic rearrangements, typically defined as variants larger than 50 base pairs (bp) in length [1]. They can affect millions of nucleotides in the human genome, thereby directly or indirectly influencing molecular and cellular processes [2], functional consequences [3], individual phenotypes [4], 3D structure [5], and susceptibility to various diseases [6]. In terms of their distribution in humans, SVs can be broadly classified into two main categories: inherited variants and somatic mutations [7]. Inherited variants are transmitted from parental genomes and constitute the primary focus of traditional genetics studies. In contrast, somatic mutations are present only in a subset of cells and exhibit a mosaic distribution, which is of particular significance in cancer genomics. Focusing on both categories of SVs remains a hotspot theme in current research.

Long‐read sequencing has proven to be a transformative technology for large‐scale genomic studies [8, 9], including genome assembly, haplotype phasing, and variant calling. Given its higher throughput and longer read lengths [10, 11], long‐read sequencing offers unprecedented opportunities for comprehensive SV detection. Recently, numerous tools have been developed for SV detection using long‐read sequencing data. These detection tools typically identify SVs from sequencing read alignments, such as cuteSV [12, 13], Sniffles2 [14], svim [15], Sawfish [16], deBreak [17], SVision‐pro [18], SVDSS [19], and Kled [20]. As each of these methods has distinct characteristics, one approach is to ensemble different callers to generate high‐confidence SV loci and then apply force‐calling methods to obtain genotypes. Such ensemble tools include CombiSV [21], SURVIVOR [22], Jasmine [23], and Truvari [24], and force‐calling tools include cuteFC [25], Sniffles2 [14], and SVJedi [26]. To reach the upper limit of SV calling, assembly‐based methods offer an opportunity by generating high‐quality assembled contigs, even at the chromosome (chr) level. These methods, such as svim‐asm [27], PAV [8], and hapdup [28], often deliver great performance but require substantial sequencing and computational resources. In the context of somatic mosaicism, Sniffles2 introduces a mosaic module to detect low frequency SVs, whereas Severus [29] and SAVANA [30] detect somatic SVs from matched tumor‐normal pairs. Although each method has its own strengths and limitations, all have contributed considerably to improving the accuracy and comprehensiveness of SV detection.

The enhanced precision of long‐read sequencing now facilitates the resolution of SVs at the haplotype level. Such haplotype‐resolved detection is pivotal for both fundamental biological research and clinical diagnostics [8]. Specifically, phased haplotypes enable the investigation of allele‐specific effects [31]; for instance, an SV on one haplotype may disrupt a transcription factor binding site within a cis‐regulatory element while the homologous allele remains functional. This distinction is crucial for elucidating the mechanisms by which SVs drive phenotypic variability and disease susceptibility. Clinically, haplotype‐resolved data are indispensable for diagnosing recessive disorders arising from compound heterozygosity [32, 33]. Distinguishing whether two pathogenic variants occur in trans (affecting both alleles) or in *cis* (affecting the same allele) directly dictates clinical interpretation. Generally, these downstream applications underscore the necessity of not only identifying precise SV patterns but also providing reliable haplotype‐resolved information.

Currently, an increasing number of variant calling methods are leveraging read phasing information to enhance variant detection accuracy, improve genotyping, and generate haplotype‐resolved variant call sets. For example, the representative single nucleotide variant (SNV) caller, Clair3 [34], integrates read phasing into its framework to amplify haplotype‐specific mutation signals and reduce systematic errors. A similar trend is emerging in the field of SV detection. Duet [35], an SV calling tool optimized for Oxford Nanopore Technologies (ONT) data, incorporates single nucleotide polymorphism (SNP)‐derived haplotype information to improve the performance of current SV detection tools. Similarly, HapKled [36] utilizes haplotype‐tagged reads to adjust clustering conditions based on read haplotypes and applies filtering strategies informed by haplotype quality. Moreover, Severus, a specialized somatic SV detector, additionally uses phasing to distinguish paternal and maternal haplotypes, thereby improving its detection ability in tumor samples. These developments highlight that the effective integration of phasing information substantially improves the accuracy and reliability of mutation detection, particularly for SV calling.

However, current phasing‐based SV‐calling methods face several inherent limitations. First, some tools are restricted to specific sequencing platforms, limiting their broader applicability. For example, Duet and HapKled are specifically optimized for ONT data, and their performance on alternative platforms remains unexplored. Second, several methods require built‐in phasing steps within their pipelines, which can be computationally intensive and time‐consuming. This requirement constrains users by preventing them from using prephased alignments or integrating customized phasing strategies. Third, many existing methods either overlook or underutilize haplotype information, making it difficult to accurately resolve multiallelic SVs or mosaic SVs. In these cases, SV signatures are often weak or highly similar across alleles, leading to ambiguous or incomplete detection. Collectively, these limitations hinder the consistent generation of high‐quality, haplotype‐resolved SV call sets.

To address these limitations, we propose cuteHap, a haplotype‐aware SV detection method that utilizes phased alignment reads to identify SVs at the germline or mosaic level and assign their corresponding phased genotypes. Phased alignment data from multiple long‐read sequencing platforms are accepted. cuteHap evaluates the genome based on phasing quality and categorizes it into high‐ and low‐quality regions, applying tailored strategies for each. In high‐quality regions, cuteHap performs haplotype‐specific, self‐adaptive clustering and refines genotypes to optimize phased assignments. In low‐quality regions, it applies a cluster credibility‐prioritized beam search approach combined with a modified Bayesian estimation framework to infer genotypes. Evaluations on both simulated and real datasets demonstrate that cuteHap delivers superior performance in SV detection and genotype assignment compared to state‐of‐the‐art methods, underscoring its effectiveness in haplotype‐aware SV analysis.

## 2 Results

### 2.1 Overview

**FIGURE 1:**
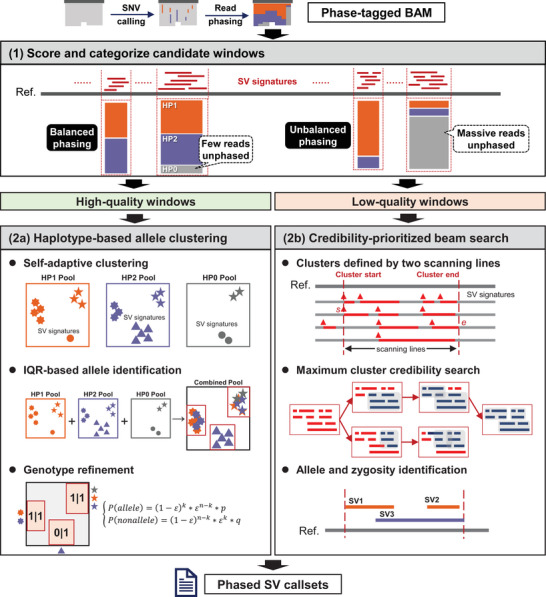
Schematic illustration of the cuteHap approach. (Step 1) SV signatures from the phased alignment are collected and preclustered as candidate windows. Each candidate window is then scored and categorized into high‐ and low‐quality windows based on the phasing status. (Step 2a) Respective self‐adaptive clustering of each haplotype and genotype refinement are applied in the high‐quality candidate windows to detect SVs. (Step 2b) Cluster credibility‐prioritized beam search and genotype likelihood estimation are applied in the low‐quality candidate windows to detect SVs.

cuteHap is a haplotype‐aware SV detection method that leverages haplotype‐tagged alignments and a reference genome to generate haplotype‐resolved SV call sets. By fully exploiting the advantages of haplotype‐tagged data, cuteHap enables accurate identification of SVs with haplotype resolution. The workflow consists of four major stages (Figure 1). First, candidate SV signatures, including clipped‐read signatures and raw‐read coordinates, are extracted using a modified signature extraction module adapted from cuteSV. Each signature is tagged with a haplotype identifier (0, 1, or 2), indicating the haplotype origin of its supporting read. Next, candidate signatures are sorted and preclustered into candidate windows, which are then scored based on phasing statistics to classify them as high‐ or low‐quality regions (Figure S1). In the third stage, high‐quality windows undergo haplotype‐specific, adaptive clustering followed by genotype refinement, whereas low‐quality windows are processed using allele identification based on cluster credibility‐prioritized beam search, combined with genotype likelihood estimation (Figure S2). The primary distinction between these two strategies lies in their dependence on phasing accuracy. In high‐quality regions where phasing is reliable, the algorithm leverages this information to perform haplotype‐specific clustering. Conversely, in low‐quality regions where phasing is less trustworthy, it employs a beam search approach across all haplotype‐tagged alignments to reduce reliance on imprecise phasing. Both strategies yield haplotype‐resolved candidate SVs. Finally, clipped‐read signatures are revisited for all candidate SVs, and each SV is classified as either a germline or mosaic variant based on its supporting read‐coverage profile.

cuteHap fully leverages phasing information in conjunction with an upgraded detection algorithm. Its key innovations are reflected in three major aspects.

First, though phasing information significantly enhances SV detection, its quality depends on both SNP calling and read phasing, and is therefore a fluctuating variable. To address this instability, cuteHap evaluates the phasing quality within each candidate window and adjusts the reliance on phasing information accordingly during downstream clustering. This prescoring mechanism allows for the optimal use of high‐confidence phasing while minimizing the impact of unreliable regions.

Second, phasing brings additional insights for accurate SV identification, greatly facilitating the detection of resolution‐limited SVs. In the human genome, many similar heterozygous variants occur at overlapping or identical coordinates. The similarity in their alignments increases the difficulty of distinguishing them. Meanwhile, fragmented alignments, often caused by sequencing errors or mapping artifacts, also make it difficult to restore complete SV signatures. cuteHap performs haplotype‐aware clustering to distinguish closely similar multiallelic SVs as well as integrate fragmented SV signatures. These methodological advances improve SV detection in terms of both resolution and accuracy.

Third, using available phasing information, cuteHap introduces a mosaic module that enables the detection of low‐frequency SVs. These SVs are difficult to identify because they have limited valid signatures in alignments, and subtle true signals are easily obscured by sequencing noise. Because the reads carrying the authentic signature of a mosaic SV tend to cluster in a single haplotype, whereas the artifacts tend to distribute separately across parental haplotypes [30], cuteHap identifies, after SV‐signature clustering, candidates that are clustered in a single haplotype but lack sufficient supporting signatures to be called heterozygous SVs. These candidates are recorded as mosaic SVs.

These designs enable cuteHap to achieve outstanding performance in haplotype‐aware SV detection, as demonstrated in the following sections.

### 2.2 High‐Performance of cuteHap in Germline SV Detection

**FIGURE 2:**
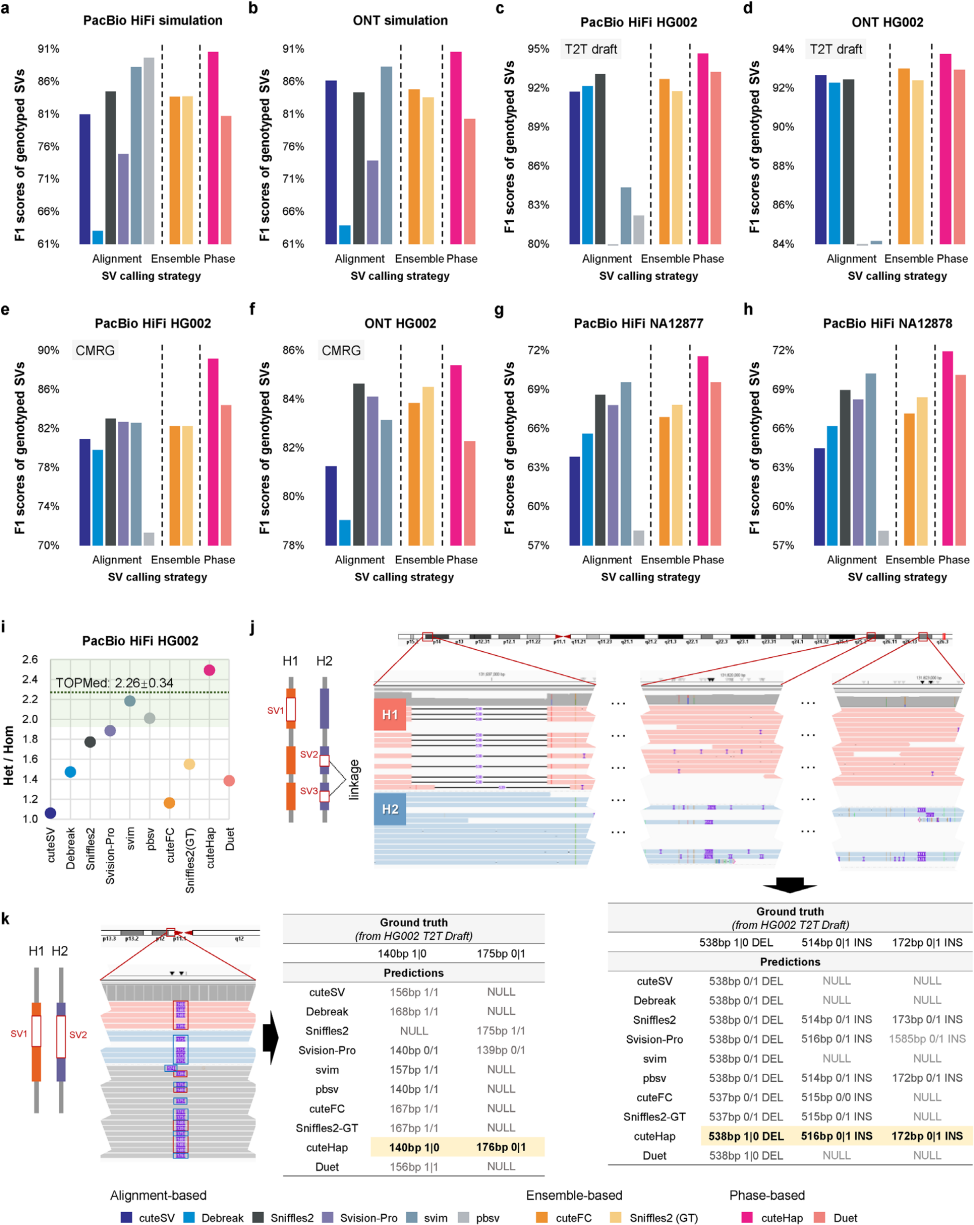
Benchmark results of SV detection on the simulated and real datasets. (a) The F1 score of different tools on simulated PacBio HiFi datasets. (b) The F1 score of different tools on simulated ONT datasets. (c) The F1 score of different tools on PacBio HiFi datasets. The ground truth is based on the T2T draft. (d) The F1 score of different tools on ONT datasets. The ground truth is based on the T2T draft. (e) The F1 score of different tools on PacBio HiFi datasets. The ground truth is based on the Genome in a Bottle (GIAB) Challenging Medically Relevant Gene Benchmark (CMRG) v1.00. (f) The F1 score of different tools on ONT datasets. The ground truth is based on the CMRG v1.00. (g) The F1 score of different tools on NA12877 PacBio HiFi datasets. (h) The F1 score of different tools on NA12878 PacBio HiFi datasets. (i) The proportion of heterozygous SVs and homozygous SVs detected by different tools. The dashed line represents the proportion observed in Trans‐Omics for Precision Medicine (TOPMed). (j) An example of heterozygous SV linkage. cuteHap, as a phasing‐based tool, distinguishes the haplotype differences of heterozygous SVs and correctly identifies these SVs. (k) An example of two heterozygous SVs that are detected correctly only by cuteHap.

We selected a total of ten methods for comparison: deBreak, cuteSV, Sniffles2, SVision‐pro, svim, pbsv, cuteFC, Sniffles2‐GT, Duet, and cuteHap (more details in the Methods). First, we evaluated the methods on Pacific Biosciences High‐Fidelity sequencing (PacBio HiFi) and ONT simulated datasets (Table S1). cuteHap achieved high precision and recall on both simulated datasets, outperforming the second‐best tools by approximately 2–4% in recall. As a result, cuteHap yielded the best F1 scores (F1 score: 90.55% for PacBio HiFi, 90.56% for ONT, Figure 2a,b and Table S2). Notably, different tools showed platform‐specific performance preferences: pbsv ranked second on the PacBio HiFi dataset (F1 score: 89.68%), and svim held this position on the ONT dataset (F1 score: 88.33%). This suggests that some tools are optimized for specific sequencing platforms. In contrast, cuteHap consistently delivered top‐tier performance across both platforms, with the F1 scores differing by less than 0.1% between them. Its integration of phasing information enables effective haplotype allele recognition, thereby enhancing detection accuracy and sensitivity, particularly in genotype assignment.

Considering the inherent limitations of simulation, we then selected real sequencing data from the globally studied Ashkenazi sample HG002, utilizing both PacBio HiFi and ONT datasets. First, we used the official benchmark commands to assess performance against a high‐confidence ground truth generated by the Telomere‐to‐Telomere (T2T) Q100 project (https://github.com/marbl/hg002). Among the ten evaluated tools, cuteHap achieved the highest F1 scores on both PacBio HiFi and ONT datasets (F1 score: 94.66% for PacBio HiFi, 93.75% for ONT, Figure 2c,d and Table S3). Between these two datasets, performance on PacBio HiFi surpassed that of ONT by approximately 1%, attributable to the higher base accuracy of HiFi sequencing (Table S1). However, this benchmark applies a relatively lenient criterion considering true‐positive SV genotypes based on the allele‐count mode (“AC”), rather than exact genotype concordance. To address this, we further implemented a stricter benchmark mode (“Single”), which matches detected SVs individually against the corresponding calls in the ground truth, requiring both precise spatial coordinates and accurate genotypes for true‐positive classification. Under this more rigorous evaluation, the performance of all tools declined markedly, reflecting the challenges of distinguishing and genotyping SVs in multiallelic and highly repetitive regions. Notably, cuteHap maintained the highest F1 scores (73.29% for HiFi, 70.55% for ONT), outperforming other methods by over 6% and 3% (Figure S3 and Table S4), demonstrating its high performance and platform‐independent robustness.

To further challenge the methods in medically relevant contexts, we applied a benchmark from the Genome in a Bottle (GIAB) consortium, comprising 216 SVs across clinically important genes. cuteHap successfully identified most true‐positive SVs in these regions and attained the highest F1 scores on both sequencing platforms (89.15% on PacBio HiFi and 85.39% on ONT; Figure 2e,f and Table S5). These superior results can be attributed primarily to cuteHap's haplotype‐aware clustering algorithm, which maximizes the recognition of SV signatures in various alleles and further enhances allele classification, enabling the distinction of closely spaced or multiallelic SVs that are often challenging to resolve.

To ensure the robustness of our method and mitigate potential overfitting to specific samples, we further evaluated the detection performance of each tool on additional samples from the Platinum Pedigree Consortium [37] and Human Genome Structural Variation Consortium [8]. Using the high‐confidence call sets released by the consortium as reliable baselines for benchmarking, cuteHap achieved the highest F1 scores on both NA12877 and NA12878 individuals, and consistently ranked among the top‐performing methods on the HG00096 and HG01505 individuals (Figure 2g,h and Table S6). Similarly, Duet, as another phasing‐based method, also performed at a top level, further demonstrating the advantages of leveraging phasing information in SV detection. Together, these results demonstrate the robust detection capability of cuteHap.

In addition, we analyzed the ratio of heterozygous to homozygous SVs in the detected HG002 and compared these ratios to those reported in well‐characterized global cohorts [38]. Figure 2i shows that cuteHap achieved a heterozygous‐to‐homozygous (het/hom) ratio of 2.49, making it one of the three tools with values falling within the Trans‐Omics for Precision Medicine (TOPMed) reported interval (2.26 ± 0.34). Other methods yielded ratios below 1.89 (Table S7), indicating a relative deficiency in detecting heterozygous SVs. This deficiency mostly arises from misclassifying multiple adjacent heterozygous SVs as single homozygous SV, which considerably diminishes the power to resolve heterozygous variants.

Phasing allows cuteHap to resolve linkage between heterozygous SVs. As shown in Figure 2j, three heterozygous SVs are located within chr10: 131 696 600–131 823 000, with two insertions residing on the same haplotype, indicating variation linkage, and a deletion present on the alternate haplotype. Under haplotype‐aware clustering, cuteHap accurately detects all three SVs according to their haplotype, and assigns their phased genotypes to uncover their linkage relationships, whereas other tools struggle not only to detect these heterozygous SVs but also to infer their correct haplotype linkage.

In addition, phasing provides the opportunity to better distinguish multiallelic SV alleles. Figure 2k depicts a multiallelic locus on chr1: 3 212 589, where two heterozygous insertions of 140 and 175 bp occur. In the Integrative Genomics Viewer (IGV) snapshot, insertion signatures cluster distinctly around these two sizes. Although some reads in this region lack phasing information (haplotype tag 0), all reads supporting the 140 bp insertion are phased as haplotype 1, while those supporting the 175 bp insertion are phased as haplotype 2. Using a cluster credibility‐prioritized beam search, cuteHap successfully identifies both insertions with accurate lengths and correct zygosity. In contrast, other methods either report a single homozygous insertion approximating the average length of the two variants or incorrectly assign two heterozygous insertions with inaccurate sizes. Collectively, these benchmarks demonstrate the robust SV detection and phased genotype assignment capabilities of cuteHap in germline individuals.

### 2.3 Robust Performance of cuteHap in Diverse Genomes

**FIGURE 3:**
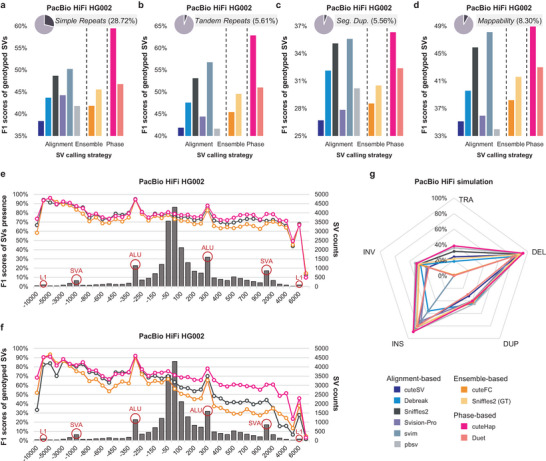
Overall results in different aspects of SV detection. (a) The F1 score of different tools in simple repeat regions on PacBio HiFi datasets. (b) The F1 score of different tools in tandem repeat regions on PacBio HiFi datasets. (c) The F1 score of different tools in segmental duplication regions on PacBio HiFi datasets. (d) The F1 score of different tools in low‐mappability regions on PacBio HiFi datasets. (e,f) The F1 presence score (e) and F1 genotype score (f) of different tools, along with the counts of SVs under different SV length intervals, on PacBio HiFi datasets. Only three methods are shown, representing the best tools among alignment‐based, ensemble‐based, and phasing‐based methods, respectively. (g) The F1 score of different tools on different SV types (deletion (DEL), insertion (INS), inversion (INV), duplication (DUP), and translocation (TRA)) in simulated PacBio HiFi datasets.

Repeat regions constitute approximately 50% of the human genome [39] and are functionally important, being associated with numerous genes and diseases [9]. Although long‐read sequencing has substantially advanced analysis within these regions, the precise extent of its advantages remains unclear. To investigate this, we conducted a detailed analysis of SV detection performance across different repeat types. Specifically, we extracted four genomic categories: simple repeats, tandem repeats, segmental duplications, and low mappability regions, and evaluated the performance of each method within these regions. As shown in Figure 3a–d, cuteHap exhibited notably high performance, particularly in simple and tandem repeat regions, achieving F1 scores more than 9% and 6% higher than the second‐best tool, svim. Detection within segmental duplication regions proved more challenging, because of the long repetitive units and variable copy numbers across individuals [9]. Although cuteHap maintained the highest F1 scores in these regions, its performance declined considerably compared to other regions, indicating that segmental duplications remain a difficult genomic context requiring further methodological improvements. Additionally, in low mappability regions, cuteHap also outperformed other tested tools, achieving the highest F1 scores, thereby demonstrating robust detection capability across challenging genomic landscapes (Table S8).

Subsequently, we assessed detection performance across different SV size ranges. The histograms in Figure 3e,f illustrate the distribution of SV counts by SV size. Consistent with previous studies highlighting the widespread presence of mobile elements in the human genome [40], cuteHap exhibited prominent peaks around 300, 1000, and 5000 bp, corresponding to the typical sizes of common mobile elements (i.e., Alu element, SINE‐VNTR‐Alu element, and LINE‐1 element). We then selected representative best‐performing tools from three categories: Sniffles2 (alignment‐based), cuteFC (ensemble‐based), and cuteHap (phasing‐based), and evaluated their F1 scores across varying SV sizes (Table S9). As shown in Figure 3e, cuteHap consistently outperformed the others irrespective of SV size, with particularly strong performance for insertions ranging from 100 to 3000 bp. When considering genotype accuracy, the performance of all tools decreased markedly, especially for insertions; however, cuteHap's advantage became even more pronounced (Figure 3f). This decline likely reflects the challenges in correctly assigning genotypes within multiallelic and repetitive regions, which are exacerbated for insertions due to the presence of multiple heterozygous insertions sharing similar genomic coordinates but distinct alleles, leading to misclassification. By leveraging phasing information, cuteHap performs allele clustering separately within each haplotype, enabling effective discrimination of heterozygous insertions and facilitating the accurate detection of multiallelic SVs.

Subsequently, we evaluated tool performance across different SV types. For insertions and deletions in HG002, cuteHap achieved the highest overall performance among all tools (Figures S4 and S5 and Tables S10 and S11). To further assess performance across a broader range of SV types, we conducted simulated genomes including five SV types and generated PacBio HiFi‐based sequencing data. cuteHap consistently achieved high F1 scores in all categories (Figure 3g and Table S12). Among the five SV types, deletions and insertions were the most accurately detected, with cuteHap achieving the highest F1 scores (93.68% and 89.31%). In contrast to them, the detection accuracy for inversions, duplications and translocations was markedly lower. This decline can be attributed to the inherent complexity of these variants, which poses challenges for accurate alignment and phasing of duplicated or translocated alleles. Despite this, cuteHap maintained the highest performance across translocations, and also ranked among the top methods for inversions and duplications. These results underscore cuteHap's robustness and versatility in detecting a wide range of structural variants.

In addition, we evaluated SV detection tools on two widely studied trios, including the Ashkenazim trio and the Platinum Pedigree trio, using Mendelian inheritance principles as an indirect benchmark, measuring compliance with these inheritance patterns. Traditional Mendelian analyses typically focus only on the false discovery rate of violations, neglecting the overall discovery capacity within the trio. To address this, we introduced a novel metric, trio Mendelian concordance rate (tMCR), defined as the harmonic mean of the true discovery rate and discovery performance. As shown in Figure S6, cuteHap achieved the highest tMCR, outperforming other methods by approximately 5% and 2% on the Ashkenazim and Platinum Pedigree trios, respectively, indicating superior SV discovery in trio contexts (Table S13). Figure S7 highlights a region on HG002 containing two heterozygous insertions (4030 and 5187 bp) on chr1: 236 097 300, inherited from the paternal and maternal parents, respectively. cuteHap enhances signature extraction and recalls clipped reads surrounding the breakpoints, which facilitates the identification of these large‐sized SVs. Notably, only cuteHap and SVision‐pro identified both insertions in HG002; however, only cuteHap detected the 5187 bp insertion in HG004, accurately reflecting Mendelian inheritance patterns.

### 2.4 How Does Phasing Influence SV Detection in cuteHap

**FIGURE 4:**
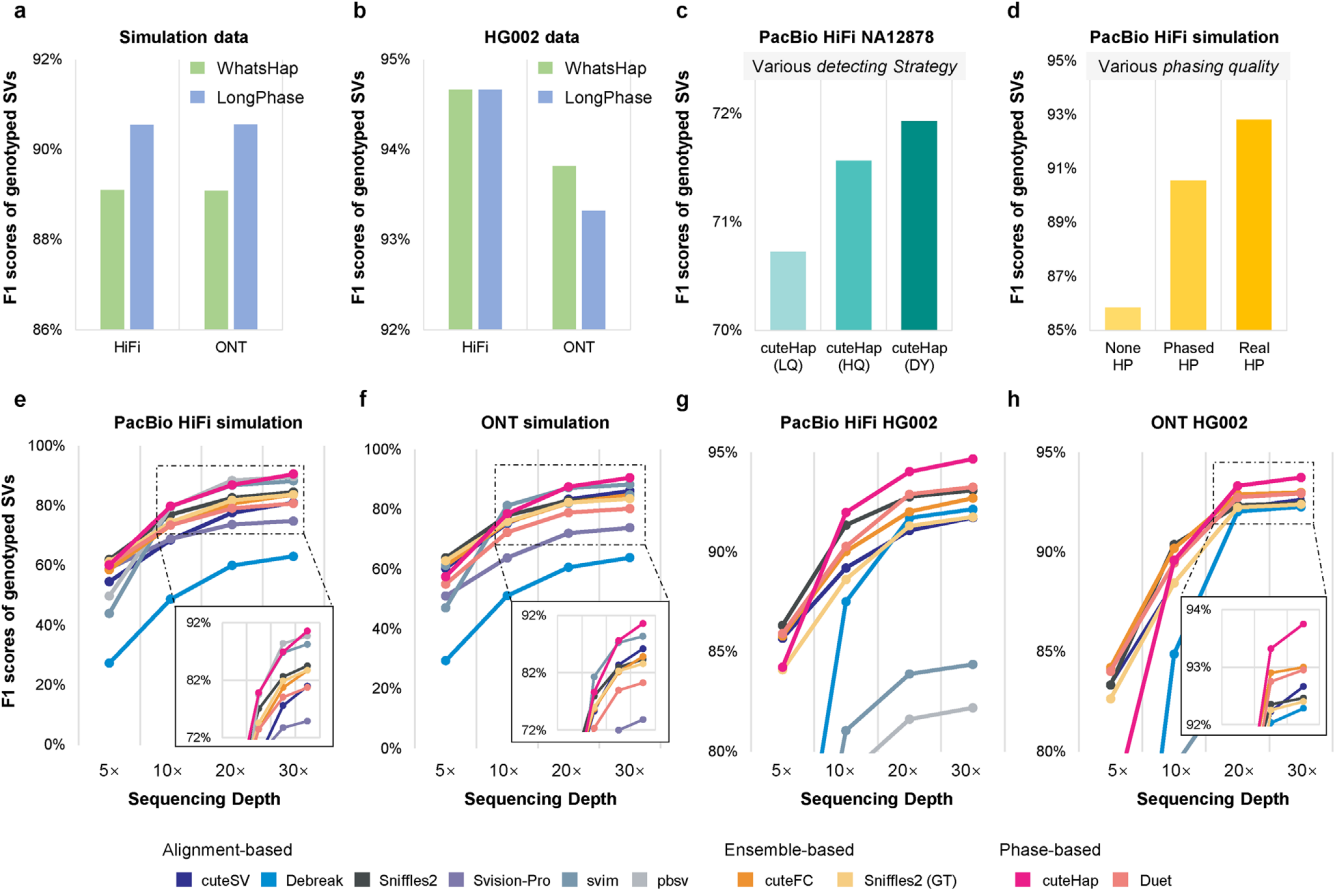
Benchmark results of additional factors in cuteHap. (a) The F1 score of cuteHap using different phasing tools on simulated datasets. (b) The F1 score of cuteHap using different phasing tools on HG002 datasets. (c) Ablation experiments under different detection strategies. (d) Ablation experiments under different phasing quality levels. (e) The F1 score of different tools under varying sequencing coverages on simulated PacBio HiFi datasets. (f) The F1 score of different tools under varying sequencing coverages on simulated ONT datasets. (g) The F1 score of different tools under varying sequencing coverages on PacBio HiFi datasets. The ground truth is derived from the T2T draft. (h) The F1 score of different tools under varying sequencing coverages on ONT datasets. The ground truth is derived from the T2T draft.

This section analyzes additional factors that influence the performance of cuteHap. First, read phasing based on SNV calling, as the upstream procedure of cuteHap, directly influences the performance of SV detection. We applied different phasing methods (i.e., LongPhase [41] and WhatsHap [42]) on both simulation and real datasets to evaluate the influence of reads phasing. Figure 4a,b and Figure S8 indicate that cuteHap maintains highly stable detection performance when employing different phasing methods. In simulated datasets, cuteHap showed a F1 score variation of approximately 1% between two different phasing inputs. However, when it comes to real datasets, the F1 scores of cuteHap only varied by no more than 0.5% across a range of sequencing platforms and diverse samples (Table S14). This stability underscores the robustness of cuteHap with respect to the choice of phasing tools.

Next, the sequencing coverages are also known to be an important influence factor of SV detection. We performed random downsampling to explore the influence of the sequencing coverages on SV detection. On both simulation and real datasets of low coverage, the performance of each tool declines with decreasing coverage (Figure 4e–h). Specifically, when the coverage decreased to 20×, the performance of cuteHap remained the best and largely stable. When decreasing to 10×, the decline is more apparent but cuteHap still maintained the overall best performance. However, with further decrease, a noticeable decline in performance was observed in cuteHap, particularly for ONT datasets. We observed that this sharp performance drop is caused by the accuracy of SNP calling, a fundamental prerequisite for read phasing. As shown in Figure S9, the F1 scores of SNP calling have a pronounced reduction at 5× coverage in ONT datasets (only 69.14%, Table S15), which hampers the confidence of read phasing and ultimately limits the support of these phase‐based methods. This phenomenon is also consistent with other studies on bioinformatics tools and global genomics research [37], which continuously recommend sequencing coverage to be no less than 10×.

To validate the effectiveness of cuteHap's implementation, we conducted an ablation experiment designed to benchmark the contribution of its dynamic (DY) strategies and explore the superior limit of the strategies. In the cuteHap framework, the genome is scored to differentiate high‐quality (HQ) and low‐quality (LQ) regions, enabling the application of different detection strategies accordingly. In this experiment, we compared three variants of the method: (i) the full version using DY strategies (default in cuteHap), (ii) a version applying only HQ strategies across all regions, and (iii) a version applying only LQ strategies. As shown in Figure 4c, the dynamic approach yielded the highest F1 scores, confirming the necessity and effectiveness of adapting strategies to local phasing quality (Table S16).

Furthermore, leveraging the availability of ground‐truth haplotypes in simulated datasets, we examined the influence of phasing accuracy on SV detection. To this end, we prepared two modified alignment inputs: one with all phasing tags removed, and another in which reads were retagged based on their true haplotypes from the simulation (Figure 4d and Table S16). Compared to the unphased input, cuteHap achieved substantially improved performance when using phased alignments, highlighting the critical role of phasing information. The best performance was observed with alignments retagged using the true haplotypes, highlighting the potential of perfect phasing to eliminate errors introduced by SNP calling and phasing processes, thereby greatly enhancing SV detection accuracy. Although such ideal phasing is unattainable in real‐world datasets, cuteHap maximally exploits available phasing information. This ability enables it to consistently deliver superior performance across various conditions in simulated datasets, further validating the robustness and adaptability of the method.

### 2.5 Detection of Mosaic SVs Reveals Somatic Mosaicism

**FIGURE 5:**
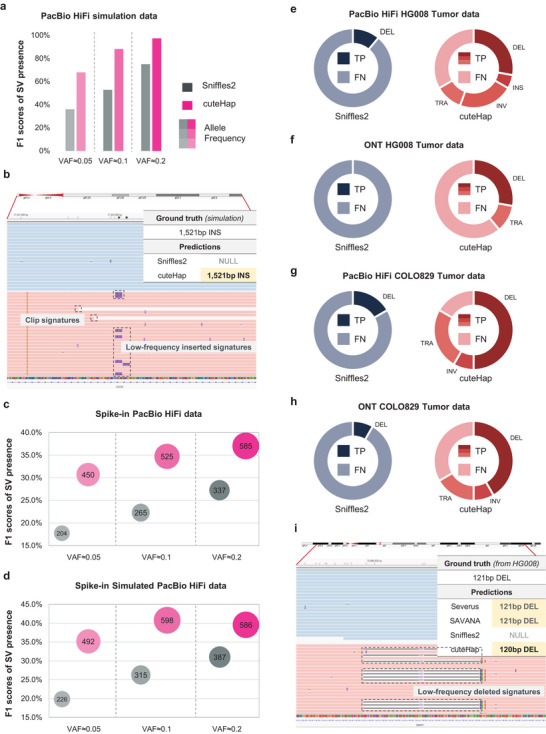
Benchmark results and examples of mosaic SV detection. (a) The F1 score of different tools for mosaic SVs on simulated PacBio HiFi datasets. (b) An example of a mosaic insertion (VAF≈20%) detected only by cuteHap. (c) The F1 score and True Positive (TP) counts of mixed PacBio HiFi data under different VAFs. (d) The F1 score and TP counts of mixed simulated data under different VAFs. (e) Detection results of HG008 on PacBio HiFi datasets. Dark colors represent true positive SVs of different SV types, and lighter colors represent false negative SVs. (f) Detection results of HG008 on ONT datasets. (g) Detection results of COLO829 on PacBio HiFi datasets. (h) Detection results of COLO829 on ONT datasets. (i) An example of a low‐frequency tumor deletion in HG008 detected only by cuteHap and other somatic‐specific detection tools.

Except for germline SVs, which exist in human genomes from the zygote, cells also acquire variations in their Deoxyribonucleic Acid (DNA) sequence after fertilization, which are known as somatic mosaicism. However, because these SVs are only present in a subset of cells, they typically occur at lower variant allele frequency (VAF) and are difficult to detect. To address this issue, cuteHap incorporates a mosaic detection module specifically designed to identify SVs with low VAFs. To evaluate its effectiveness, we first simulated three datasets containing SVs with VAFs ranging from 5% to 20%. As shown in Figure 5a, compared with Sniffles2 which also includes a mosaic module, cuteHap consistently detects more true‐positive mosaic SVs and demonstrates high accuracy (>97%) across various VAFs. When VAFs are ≥20%, cuteHap also achieves a high recall rate (96.86%), resulting in a notably high F1 score. As VAF decreases, detection becomes more challenging due to the limited number of supporting signatures, which are more easily confused with false positives. Consequently, whereas the recall rate declines for all tools, cuteHap continues to achieve the highest recall and F1 scores (Table S17). An illustrative example in Figure 5b presents a mosaic insertion with a VAF of 20%. After applying clustering to the extracted insertion and clipped signatures, cuteHap recognizes that although the signatures can be clustered into one candidate, they are insufficient to support this SV as heterozygous. Consequently, the mosaic module classifies this event as a mosaic SV.

Considering that the simple simulation still lacks authenticity and robustness, we downsampled the HG002 alignments to an allele frequency of 5%–20% and spiked them into an existing sequencing dataset of the NA12878 sample, to simulate the existence of low‐frequency alleles. When applying the mosaic detection in this spike‐in sample, both methods reported the low‐frequency SVs that correspond with the HG002 ground truth. cuteHap achieves higher F1 scores, together with more true‐positive SVs (Figure 5c and Table S18). However, the F1 scores are slightly lower than those obtained from direct detection of HG002. The main reason is that, although we extracted the alignments from one haplotype as far as possible, the randomly downsampled reads confused reads from the other original haplotype. In addition, mosaic SVs exist in the NA12878 sample. These preexisting mosaic SVs influenced the evaluation of low‐frequency SVs carried only by the spike‐in HG002. Therefore, we further simulated a sequencing dataset for NA12878 containing only its known homozygous and heterozygous SVs, and spiked the downsampled HG002 datasets into this simulated dataset. In the new dataset, the F1 scores of both methods improved (Figure 5d and Table S18), and cuteHap still achieves the best performance.

A well‐studied category of mosaic SVs is cancer‐associated somatic SVs, which are characterized by complexity and heterogeneity, containing a large number of low‐frequency SVs. Herein, two real tumor samples (i.e., HG008 and COLO829) were selected to evaluate the performance of cuteHap in detecting low‐frequency SVs in tumor genomes. To establish ground truth sets, we manually extracted the low‐frequency SVs in their high confidence call sets by visually examining each candidate in IGV screenshots and confirming a low VAF. On both tumor datasets under PacBio HiFi and ONT sequencing, cuteHap identified more low‐frequency SVs with a greater variety of SV types (Tables S19 and S20). Specifically, cuteHap successfully detected 12 true‐positive SVs, including 5 deletions, 1 insertion, 4 inversions, and 2 translocations in the PacBio HiFi HG008 tumor sample, whereas the mosaic mode in Sniffles2 only reported 2 deletions. The successful detection of low‐frequency SVs was based on tracing the supporting signatures. Figure 5i illustrates an example of a somatic deletion in the HG008 ground truth, where both manual inspection and two somatic‐specific tools (i.e., Severus and SAVANA) reported this variant as a somatic deletion with low frequency (<0.5). In the IGV snapshot of the example, the deletion signatures are largely similar and clustered as a candidate. However, when tracing the haplotype of these signatures, they were all from haplotype 2, and their frequency could not support them as heterozygous. Thus, cuteHap recognized it as a mosaic deletion and reported the corresponding result consistent with the above tools. These benchmarks on simulated and real datasets demonstrate the multifaceted advantages of cuteHap in detecting mosaic SVs, facilitating its application in resolving low‐frequency SVs in somatic tissues.

### 2.6 Computational Performance

Detection efficiency and computational speed are critical considerations in SV analysis. We benchmarked the runtime and memory usage of various SV detection tools (TABLE 1). Following sequencing, tools employ different processing workflows to generate SV call sets. Alignment‐based methods typically involve two steps, which are read alignment and SV calling. Ensemble‐based methods require four steps, including read alignment, multiple SV calling runs, SV merging, and force calling. Phase‐based methods also involve four steps, which are read alignment, SNV calling, read phasing, and SV calling. Given that most modern systems support parallel computing, we assessed tool efficiency under multithreaded conditions (16 threads). As expected, alignment‐based tools such as Sniffles2 and cuteSV had the shortest total runtime due to their simpler workflows. Notably, read alignment and SNV calling are more time‐consuming on ONT datasets than on PacBio HiFi datasets, contributing to longer overall runtimes for ONT‐based analyses. When examining only the SV calling stage, Sniffles2(GT) was the fastest, because it benefits from predefined SV loci that bypass the need for full detection, thereby minimizing computation. cuteHap, cuteSV, Sniffles2 and cuteFC followed closely, each completing SV calling within 4 min per sample (Table S21), demonstrating the overall efficiency of current SV detection tools. Furthermore, we evaluated the runtime of cuteHap under various coverages, genome sizes, and phased blocks, and observed that the runtime scales linearly with input alignment size (Figure S10 and Tables S22 and S23). Overall, cuteHap processes alignments at a stable speed of approximately 3s/GB, demonstrating efficient and consistent performance for SV detection.

Memory usage is another key factor in evaluating the practicality of SV detection tools. As the number of processing threads increases, memory consumption also rises due to greater computational demand. Under 16 threads, the majority of tools, except for deBreak and Duet, maintain memory usage within a manageable range of 3 to 6 GB, making them suitable for standard computing environments. The higher memory requirements of deBreak and Duet stem from their computationally intensive components: deBreak leverages partial order alignment, whereas Duet includes a mandatory phasing step. In comparison, cuteHap, as a lightweight tool, uses a stable memory at about 4 GB and remains well within the capacity of most modern systems, ensuring its usability in a broad range of genomic analyses and research settings (TABLE 1).

**TABLE 1:** Elapsed time and memory footprints in SV detection.

Methods	HiFi dataset		ONT dataset	
	Elapsed time^a^ (min)	Memory footprints^b^ (Gb)	Elapsed time^a^ (min)	Memory footprints^b^ (Gb)
**Alignment‐based**: Alignment + SV calling				
cuteSV	137.6 (134.1 + **3.5**)	4.9	361.7 (358.3 + **3.4**)	5.94
deBreak	258.2 (134.1 + **124.1**)	27.88	597.7 (358.3 + **239.4**)	52.31
Sniffles2	137.7 (134.1 + **3.6**)	2.73	361.6 (358.3 + **3.3**)	4.17
SVision‐pro	138.9 (134.1 + **4.8**)	3.4	364.4 (358.3 + **6.1**)	4.16
svim	156.2 (134.1 + **22.1**)	0.79	426.2 (358.3 + **67.9**)	1.32
pbsv	192.5 (134.1 + **58.4**)	7.8	NULL	NULL
**Ensemble‐based**: Alignment + ∑*SV* *calling* + Merge + Force calling				
cuteFC	273.5 (134.1 + 135.1 + 0.9 + **3.4**)	4.56	614.6 (358.3 + 251.9 + 0.8 + **3.6**)	4.47
Sniffles2‐GT	272.2 (134.1 + 135.1 + 0.9 + **2.1**)	4.98	614.3 (358.3 + 251.9 + 0.8 + **3.3**)	7.35
**Phase‐based**: Alignment + SNV calling + Phasing + SV calling				
cuteHap	250.2 (134.1 + 85.8 + 27.5 + **2.8**)	4.27	530.7 (358.3 + 140.8 + 27.8 + **3.8**)	4.23
duet	219.4 (134.1 + **85.3** ^c^)	49.17	454.6 (358.3 + **96.3** ^c^)	55.07

^a^ The elapsed time of the SV calling procedure is indicated in bold.

^b^ The memory footprint of the SV calling procedure.

^c^ The total elapsed time for Duet, which includes SNV calling, read phasing, and SV calling.

## 3 Discussion

The rapid advancement of long‐read sequencing technologies has significantly accelerated a wide range of genomic studies, including variant detection (e.g., SNVs, indels, and SVs), read phasing, and inheritance analysis. These areas of research are increasingly interdependent, as accurate SNV calling enables reliable read phasing, and high‐quality phasing enhances the resolution and accuracy of variant detection. In this context, we present cuteHap, a haplotype‐aware SV detection method that fully leverages phased alignments to enhance both the accuracy and interpretability of SV calling. Extensive benchmarking across diverse samples and sequencing platforms consistently demonstrates the accuracy, sensitivity, and robustness of cuteHap, establishing it as a powerful tool for haplotype‐resolved structural variant analysis.

With advancements in sequencing technologies and computational methods, SV detection has evolved beyond simple, easily identifiable SVs to focus increasingly on complex and challenging variants. A key source of complexity arises from highly similar SVs, which are variants that occur in close proximity but differ subtly in their structural features. Some of these SVs share identical breakpoints and appear as multiallelic SVs, whereas others exhibit the same variant length but differ in nucleotide composition. These similarities significantly complicate accurate discrimination and allele resolution, as traditional SV callers often struggle to determine whether observed differences stem from genuine allelic diversity or from sequencing noise and alignment artifacts. Recent efforts by the T2T consortium to generate a near‐complete diploid assembly of the GIAB HG002 sample (superseding GIAB v0.6) further underscore this challenge. The updated draft reveals a substantial number of closely spaced SVs (within 1 kb), including exact‐coordinate multiallelic SVs. Assembly‐based methods offer improved resolution of such complex regions owing to their capacity to reconstruct haplotype‐specific sequences; however, they remain computationally intensive and require high sequencing depth, limiting their accessibility. In contrast, cuteHap circumvents full assembly by integrating phasing information directly into the detection pipeline. This approach provides orthogonal evidence for distinguishing SV alleles across haplotypes, thereby enhancing the resolution and accuracy of complex SV detection while maintaining computational efficiency.

The key innovation of cuteHap lies in its comprehensive utilization of haplotype information, which empowers it not only to detect the majority of individual SVs but also to accurately identify a broad spectrum of complex SVs that often elude other methods. First, after thoroughly collecting both intra‐ and inter‐alignment signatures, cuteHap employs a dual‐module strategy, distinguishing between high‐ and low‐quality regions, to automatically cluster and integrate SV signatures. Haplotype information is leveraged to the greatest extent possible, not only to differentiate similar heterozygous SV alleles but also to generate haplotype‐resolved SV call sets enriched with linkage information. In addition to SV detection, cuteHap assigns highly reliable genotypes. Unlike conventional approaches that often rely on simple thresholding or basic Bayesian estimation, cuteHap introduces a novel genotype inference model grounded in the phasing context. In high‐quality phasing windows, genotypes are directly derived from the haplotype origin of supporting alleles, and in low‐quality regions, genotypes are determined using maximum likelihood estimation and then refined based on the distribution of haplotype support. This strategy effectively mitigates misclassification of zygosity, particularly in multiallelic regions, thereby significantly enhancing genotyping accuracy.

Furthermore, cuteHap enables the detection of mosaic SVs by fully leveraging phasing information. Extensive genetic variation exists within individuals among different tissues and cells, and cells steadily acquire somatic mutations throughout life, which are often described as mosaic. These variants can profoundly alter the phenotype of a cell and are implicated in a wide variety of diseases, including cancers (the best‐known example of disease arising from somatic mutation), neurological diseases and inflammatory disorders [7]. However, identifying mosaic SVs is challenging due to the limited number of supporting reads and their similarity to sequencing errors. In cuteHap, phasing information plays a crucial role by enabling the classification of reads according to haplotype and providing insight into their zygosity. When SV signatures are concentrated within a single haplotype but lack sufficient support to be classified as heterozygous, they are more likely to represent mosaic events. cuteHap takes advantage of this characteristic by incorporating a dedicated mosaic detection module, allowing for more accurate and comprehensive identification of mosaic SVs.

Despite its advantages and innovations, cuteHap also has certain limitations. First, although current phasing tools can accurately assign haplotype tags in most genomic regions, some complex and repetitive regions remain challenging for precise phasing. Enhancing the accuracy of these phasing tools would consequently improve cuteHap's performance. Additionally, effective read phasing requires adequate sequencing depth, as too low coverage (<10×) can compromise phasing accuracy, thereby negatively impacting SV detection by cuteHap. Second, widely used phasing tools such as WhatsHap and LongPhase primarily rely on SNPs to assign haplotypes. However, beyond SNPs, the human genome harbors abundant epigenomic features such as DNA methylation, which also exhibit haplotype specificity and may serve as valuable sources for phasing and downstream SV detection. Moreover, recent advances have explored phasing in complex genomic contexts, such as polyploid genomes and somatic variation. Though cuteHap facilitates the effective mosaic SV detection, the complexity of somatic mosaicism is still contrary to our expectations. Currently tailored for diploid genomes, cuteHap's future development may include expanded capabilities to address polyploid genomes (e.g., plant SVs), heterogeneous or mosaic genomes (e.g., somatic SVs), and even single‐cell genomes.

## 4 Conclusion

cuteHap enables high‐performance, haplotype‐resolved SV detection through the use of phased alignments. By integrating two automatically selected algorithms, respective self‐adaptive clustering and cluster credibility‐prioritized beam search, cuteHap fully leverages phasing information within SV detection. Benchmarking results consistently demonstrate that cuteHap achieves superior accuracy, efficiency, and robustness compared to state‐of‐the‐art methods. These strengths highlight cuteHap as a powerful tool for generating high‐quality, haplotype‐aware SV call sets. We anticipate that cuteHap will find broad applications in advanced genomic research and precision medicine.

## 5 Methods

cuteHap took a phased alignment file and a reference genome as input, and detected SVs through various strategies of signature clustering and variant genotyping. After parsing the alignments, the collected signatures were represented as multidimensional vectors. cuteHap preclustered these signatures to identify the candidate SV windows, then categorized these candidate windows into high‐quality and low‐quality windows according to their phasing status, and implemented various clustering strategies and genotype refinement in each candidate window. For high‐quality windows, cuteHap applied self‐adaptive clustering separately on different haplotypes, and adjusted the genotype around each SV coordinate via Bayesian estimation and count‐based filtration; for low‐quality windows, cuteHap applied cluster credibility‐prioritized beam search in the whole window and assigned genotypes via modified Bayesian estimation. cuteHap also recalled clipped signatures to enhance clustered signatures. The main steps of cuteHap are depicted in Figure 1.

### 5.1 Scoring and Categorizing Candidate Windows

After collecting SV signatures from the phased alignment, cuteHap scanned the whole genome and preclustered the signatures to obtain the candidate windows where the SVs might occur, then cuteHap scored each window according to the phasing status to categorize them into high‐ and low‐quality windows. Generally, phasing aimed to partition reads into two groups, which corresponded to the paternal and maternal haplotypes, respectively. This scoring system evaluated whether the phasing status in a given window accorded with this objective, that is, whether reads were successfully and balancedly partitioned into two haplotype groups. If a significant proportion of reads could not be assigned to either haplotype, or if there was a substantial difference in the number of reads assigned to the paternal versus maternal haplotype, the window was inferred to have low phasing quality. Otherwise, the window was inferred to have reliable phasing.

First, cuteHap slightly modified the signature extraction module in cuteSV to collect the candidate SV signatures. These signatures were represented as multidimensional vectors which were similar to those in cuteSV, with an additional dimension, phasing group (0, 1, or 2), which indicated whether the haplotype on which the signature occurred was unknown, the male parent, or female parent, respectively. All signatures were grouped by their SV type, chromosome of occurrence, and sorted by the genomic coordinate. cuteHap scanned through each group and collected the signatures with nearby coordinates into one candidate window, which meant the adjacent signatures were collected into one window when they satisfied:

(1) $$start_{Sig1}-start_{Sig2}<\;max\_cluster\_bias$$

where *start_Sigi_* represents the start coordinate of the *i*‐th signature, max_cluster_bias represents the parameter of the signal variability. Then, cuteHap collected all alignment reads in the candidate window via a Genome Position Scanner (GPS) algorithm in cuteFC [25].

Second, cuteHap introduced a scoring system to evaluate the phasing quality of each candidate window. The quality of the candidate windows was defined by considering these conditions: (1) the account proportion of unphased reads in the window; (2) the balance proportion of phased reads among different haplotypes in the window. Assuming that the number of unphased reads, reads of haplotype 1, and reads of haplotype 2 were *c*_0_, *c*_1_, and *c*_2_, respectively, the candidate window was regarded as high‐quality when the window satisfied one of the following conditions:

(2) $$c_{0}+\left|c_{1}-c_{2}\right|<\min\left(c_{1},c_{2}\right)$$

(3) $$\left|c_{1}-c_{2}\right|<c_{0}<max\left(c_{1},c_{2}\right)$$

where *c_i_* represents the reads that are tagged with haplotype *i* (*i*  =  1, 2) or unphased (*i*  =  0). Otherwise, the candidate window was regarded as low‐quality. Candidate windows of different quality scores were processed using different clustering strategies.

### 5.2 Self‐Adaptive Clustering and Haplotype‐Based Allele Revision (SV Detection in High‐Quality Windows)

For the high‐quality windows, cuteHap applied self‐adaptive clustering of various haplotypes and genotype refinement to achieve SV detection (Algorithm S1).

First, cuteHap grouped all signatures in the high‐quality windows by their phasing categories (unphased, haplotype 1, and haplotype 2), and sorted each phasing category by the lengths of the signatures. Then, a self‐adaptive clustering strategy from cuteFC was applied on signatures in each phasing category to obtain clusters. After pruning the clusters with sizes smaller than the minimum number of supporting reads, each remaining cluster was regarded as a candidate SV. The initial genotype of the candidate SV was identified by the phasing category of its cluster. When the cluster belonged to the unphased category, the genotype was defined as unknown (.|.), and when the cluster belonged to the haplotype 1 or haplotype 2 category, the genotype was defined as heterozygous (1|0 or 0|1, respectively).

Second, cuteHap identified the clustered alleles by a combination step. Three values were defined to represent the length distribution of a clustered allele: *length_avg_* represented the average length of the alleles in the cluster, and *border_min_* and *border_max_* represented the lower and upper boundaries of the length distribution in the cluster, respectively. The boundary (*border_min_*, *border_max_*) was calculated using the interquartile range (IQR) method:

(4) IQR=Q3−Q1

(5) $$border_{min}=Q1-1.5*IQR$$

(6) $$border_{max}=Q3+1.5*IQR$$

where *Q*1 and *Q*3 represent the 25th percentile and 75th percentile of the lengths. The IQR method was a robust statistical technique used to identify outliers in a dataset, that could eliminate extreme deviation in skewed data. Then, cuteHap iterated through the pairwise clusters from any two different phasing categories. The two clusters were combined into a new allele when they satisfied both combination conditions:

(7) $$min\left(length_{avg1},length_{avg2}\right)/max\left(length_{avg1},length_{avg2}\right)>0.9$$

(8) $$\max\left(\mathrm{borde}r_{\min1},\mathrm{borde}r_{\min2}\right)<\min\left(\mathrm{borde}r_{\max1},\mathrm{borde}r_{\max2}\right)$$

where *length*_*avg*1_ and *length*_*avg*2_ represents the average allele length of the two clusters, *border*_*min*1_ and *border*_*min*2_ represents the smallest allele length of the two clusters, *border*_*max*1_ and *border*_*max*2_ represents the largest allele length of the two clusters, respectively. For example, when the two clusters had the initial genotype “0|1” and “1|0”, they were combined to generate the new allele “1|1”; when the two clusters had the initial genotype “0|1” and “.|.”, they were combined to generate the new allele “.|1”, and so on. The clusters that could not be combined with others remained unchanged.

Third, cuteHap refined the genotypes of the candidate SV through Bayesian estimation. This step aimed to determine whether the clustered alleles were real alleles, alleles introduced by mosaic variation, or alleles generated by sequencing or alignment errors. Bayes estimation was applied using the following formula:

(9) $$\left\{\begin{array}{c} P\;\left(allele\right)={\left(1-\varepsilon \right)}^{ki}\;*\varepsilon^{ni-ki}*p\\ P\;\left(nonallele\right)={\left(1-\varepsilon \right)}^{ni-ki}\;*\varepsilon^{ki}*\left(1-p\right) \end{array}\right.$$

where *ki* represents the SV allele signatures in the *i*‐th haplotype for the candidate SV, *ni* represents the total reads in the *i*th haplotype around the candidate SV, and ε represents the probability that a read is mapped to a given zygosity erroneously. If the probability of nonexistent alleles was higher, the clustered alleles would be discarded from the clusters.

Finally, the uncertainty of SV calls was measured by computing confidence interval. The confidence intervals of SV coordinate (*CIPOS*) and SV length (*CILEN*) were calculated as follows:

(10) $$CIPOS=Z*std_{pos}/\sqrt{n}$$

(11) $$CILEN=Z*std_{len}/\sqrt{n}$$

where *std_pos_* and *std_len_* represents the standard deviation of the coordinate or length of supporting SV signatures, and *n* is the number of the supporting SV signatures, *Z* is the Z‐score corresponding to the desired confidence level (e.g., 1.96 for 95% confidence). This formula, based on the standard normal distribution, estimated the range within which the true population mean was expected to lie with specific confidence.

### 5.3 Cluster Credibility‐Prioritized Beam Search (SV Detection in Low‐Quality Windows)

For the low‐quality windows, cuteHap applied beam search to accurately cluster SV signatures across haplotypes and assigns genotypes through likelihood estimation for SV detection (Algorithm S2).

First, for each candidate window, cuteHap collected the coordinates and lengths of all the SV signatures within the window. Each signature was represented as a triple (*position*,  *length_occupy_*, *length*), where *position* indicated the genomic coordinate of the signature, *length_occupy_* indicated the number of reference bases occupied by the signature (equal to signature length for deletions, and 1 for insertions), and *length* indicated the actual length of the signature. Subsequently, a set of all possible candidate clusters was identified from the window. By traversing the coordinates of signatures, potential candidate clusters were constructed.

Next, cuteHap calculated the score of each potential candidate cluster. Signatures within the window were categorized by their read id, and the probability of each read being clustered into each candidate cluster was then computed sequentially. Assuming the current read contained sorted signatures $Sig_{i}\left(i\in \;[1,\;n]\right)$, for a candidate cluster *C*, cuteHap enumerated the coordinates of the signatures and calculated clustering score between the signatures in the enumerated interval and the candidate cluster. The similarity score between signatures (from the same read) and a candidate cluster was defined by their length similarity *Length_sim_*, coordinate similarity *Coordinate_sim_*, and signature interval *Space*. The formulas were:

(12) $$\mathrm{Lengt}h_{\mathrm{sim}}=\max\left(\frac{\min\left(l_{s},\;l_{c}\right)}{\max\left(l_{s},\;l_{c}\right)}\;*2.5-1.5,0\right)$$

(13) $${\mathrm{Coordinate}}_{\mathrm{sim}}=\max\left(1-\log_{10}\left(\left|p_{s}-p_{c}\right|+1\right)*0.25,0\right)$$

(14) $$Space=\frac{max\left(l_{c}-l_{stotal},0\right)}{l_{c}}$$

(15) $$Score\left(Sig,\;C\right)=Length_{sim}\;*Coordinate_{sim}*Space$$

where *l_s_*, *l_stotal_*, *p_s_* represents the actual length, total occupancy length, and coordinate of the signatures, respectively, and *l_c_* and *p_c_* represent the length and coordinate of the cluster. The maximum *Score* is denoted as the similarity score between the current read and the cluster.

cuteHap traversed all reads within the window to obtain the similarity score between each read and the candidate cluster *C*. The overall joint score for *C*, reflecting the credibility score for this candidate cluster, was then calculated. Let *P_i_* denote the prior credibility probability of cluster *C* given the presence of the *Read_i_*, which could be calculated as the score above. Assuming an equal prior probability for cluster credibility and independence among signatures, according to Bayes' theorem, the posterior credibility probability of the cluster *C* was derived as:

(16) $$\mathrm{Scor}e_{C}=\frac{\prod P_{i}}{\prod P_{i}+\prod \left(1-P_{i}\right)}$$

This probability served as the credibility score for cluster.

Finally, cuteHap performed a cluster credibility‐prioritized beam search across all candidate clusters (default beam_size= 20). The search state was represented by a set of clusters, with the objective of identifying a valid cluster set that maximizes both capacity and overall credibility. Initially, the search set for round 0 was defined as *Ans*_0_ =  {*S*_0_}, where *S*_0_ =  ∅. The algorithm proceeded iteratively. In round *i*, for each cluster set *S* in the previous round *Ans*_*i* − 1_, a new set *S*′ was generated by adding a candidate cluster to *S*. After verifying its validity, *S*′ was added to the preliminary set *PreAns_i_*. For each *S*, the number of newly generated sets did not exceeded beam_size/2. Then, the credibility score of the generated sets was calculated through a dynamic programming algorithm optimized for interval‐matching scenarios (Cluster‐matching Dynamic Programming). The cluster sets with top beam_size scores were selected to form *Ans_i_*. The dynamic programming aimed at maximizing the credibility score in forming the cluster set. Let *f*_*i*, *j*_ denotes the clustering similarity between *Sig*_1_ to *Sig_i_* and clusters *C_i_* to *C_j_*, where:

(17) $$\begin{array}{ccc} f_{i,\;j} & = & \max\left(\max(f_{i-1,\;j},f_{i,\;j-1}\right),\;f_{k,j-1}\\ & & +Score\left(Sig_{p∼i},\;C_{j}\right))\left(0<k<p\leq i\right) \end{array}$$

A cluster set *S* is considered valid only if it satisfies all the following conditions:

Condition 1: The similarity between any two clusters in *S* was smaller than 0.5.

Condition 2: In the optimal credibility‐based assignment for *S*, each cluster was matched to at least 2 signatures.

Condition 3: Considering the characteristics of a diploid genome, at most two clusters from *S* could exist at the same position.

Here, condition 3 was verified by scanning the start and end coordinates of all clusters in *S*. A counter was incremented by 1 upon encountering a start coordinate of cluster and decremented by 1 upon encountering an end coordinate. If the counter exceeded 2 at any point, it indicates three clusters would coincide at that position, violating the diploid assumption and rendering the set invalid.

The beam search terminates at round *k* when *Ans_k_* became empty. The cluster set with the highest credibility in *Ans*_*k* − 1_ was selected, and each cluster in this set was represented as a detected SV. Reads containing the signatures in the cluster were recorded as alternative reads, and reads without supporting signatures were recorded as reference reads. The genotype of the SV was assigned through maximum likelihood estimation.

### 5.4 Recalling Read Clip Signatures

The signatures of insertions in cuteHap could be mainly divided into two categories: intra‐alignment from the alignment Compact Idiosyncratic Gapped Alignment Report and interalignment from split alignments. However, considering the limited length and random distribution of sequencing reads, when large insertions occured on the genome, sequencing reads might not be able to cross the entire insertion sequence. To comprehensively recalled this kind of signature, cuteHap first records all alignment clips in the signature extraction module. Then, after obtaining the candidate SV clusters, cuteHap iterated through these clusters using a scanning line and checks whether any remaining clip signatures near the clustered SV breakpoints. These corresponding clip signatures were also added into the clusters to enhance the signatures of the detected SVs.

### 5.5 Somatic Mosaicism Identification

cuteHap identified mosaic SVs with low allele frequency using adapted models based on germline detection. The core detection principle relied on identifying those SVs that had clustered supporting signatures but whose read support was insufficient to be classified as heterozygous SVs.

The window scoring and categorizing follow the same procedure as described above. For high‐quality windows, after performing haplotype‐specific signature clustering, cuteHap measured the proportion of reads containing clustered signatures in the haplotype. When this proportion falls within the interval [0.05, 0.45], the allele in the corresponding haplotype was marked as a mosaic allele. Then, the combination across haplotypes was applied. A mosaic SV was reported if the combination results in two mosaic alleles, or only one mosaic allele remains uncombined with any other existing alleles. For low‐quality windows, the allele classification was implemented the same way as described above. The SV genotype was first calculated via maximum likelihood estimation. SVs assigned a genotype containing the assured allele were filtered out as germline SVs. For the remained SVs, it was assessed whether the proportion of reads containing SV signatures across the entire surrounding read group lied within the interval [0.05, 0.4]. If this low‐frequency criterion was met, the SV was reported as mosaic.

### 5.6 Algorithm Complexity Analysis

In the scoring and categorizing candidate windows step, the GPS algorithm was applied, which contains a sort procedure. Therefore, the time complexity of this step was O((*n* + *m*)log(*n* + *m*)), where *n* represents the number of candidate windows, *m* represents the number of reads. In the self‐adaptive clustering and haplotype‐based allele revision step, the self‐adaptive clustering traversed the signatures once, and the combined steps traverse the clusters in pair. Therefore, the time complexity of this step was O(*t* + *n* * *m*), where *t* represents the signatures in a window, *n* and *m* represents the sizes of the clusters in different haplotype. In the cluster credibility‐prioritized beam search step, the time complexity of the cluster‐matching Dynamic Programming was O(*nk*log*n*), where *n* represents the number of signatures, and *k* represents the number of clusters. And the time complexity of beam search was O(*b* * *n* ^2^ * *m*log*n*), where *b* represents the size of the beam, *m* represents the number of result clusters.

### 5.7 Implementation of Simulation, Read Preprocessing, and SV Calling

Our benchmark encompassed five types of SVs: insertion (insertion of sequence relative to the reference), deletion (deletion relative to the reference), inversion (inversion of reference sequence), duplication (Region of elevated copy number relative to the reference), and translocation (interchromosome translocations between reference). For the simulation datasets, 10 564 structural variations on chr 1 and 2, including 7594 insertions, and 2970 deletions, were first simulated. Considering the insufficient detection sensitivity of other SV types, 450 inversions, 450 duplications, and 250 interchromosomal translocations on chr 1 and 2 were further simulated. For mosaic SV simulation, 185 insertions and 102 deletions on chr 22 were simulated. Then three simulation donor genomes were constructed, respectively by modifying the reference genome GRCh38 via VISOR [43], and simulated sequencing reads of 30× based on PacBio HiFi and ONT models via pbsim3 [44].

During read preprocessing, sequencing reads were first aligned to the GRCh38 reference genome using minimap2 [45]. Subsequently, Clair3 was employed for SNV detection, and LongPhase was used for read phasing. LongPhase begins by assigning haplotype tags to the detected SNVs to generate phased SNV calls, and then labels the reads based on this haplotype information and ultimately outputs a phased alignment file.

When performing SV calling, cuteSV (v 2.1.2), Sniffles2 (v 2.2), deBreak (v 1.0.2), SVision‐pro (v 1.8), svim (v 1.4.2), pbsv (v 2.11.0), and Duet (v 1.0) were run. For the 30×, 20×, 10×, and 5× datasets, the parameter representing “minimum supporting reads” were set to 5, 4, 3, and 2, respectively.

When performing SV ensemble calling, the SV calling results were collected from cuteSV, Sniffles2, deBreak, and SVision‐pro and merged the four call sets via Jasmine [23]. The call sets were then filtered, and only the SVs that were supported by at least two callers were retained. Then we ran cuteFC (v 1.0.1) and Sniffles2 to assign genotypes for the ensembled SV call sets.

The detailed commands are provided in the Supporting Information.

### 5.8 Implementation of Spike‐In Datasets

It was mixed low coverage HG002 datasets with NA12878 datasets and generated a spike‐in sample to simulate mosaic datasets. The alignments from haplotype 1 in chr1 of the official phased HG002 datasets were selected as the basic alignments. Then the local average coverage of genomic blocks, and adaptively selected downsampling ratios were measured for each block to downsample the basic alignments to 5×, 10×, and 20×. These downsampled datasets were further merged with the 97× NA12878 datasets by samtools to simulate a mosaic frequency about 5%, 10%, and 20%.

To eliminate potential mosaic SVs in NA12878, a 90× NA12878 dataset with all SVs from the NA12878 individual from the Platinum Pedigree call sets were further simulated. This sequencing dataset was then mixed with the above downsampled HG002 to generate simulated spike‐in sequencing datasets.

### 5.9 Implementation of Benchmarking

In benchmarking the SV call sets, we used Truvari (v 5.1.1) [24] to evaluate the SV calling performance of the methods. In the stricter benchmark mode (“Single”), Truvari was equipped with parameters “‐p 0 ‐r 1000 ‐passonly”, which represents when comparing ground truth SV and detected SV, if the breakpoint distance was smaller than 1000 bp and the SV size similarity was larger than 0.7, and the genotype was exactly same in zygosity, the detected SV was marked as correct. In benchmark mode “Allele‐Count”, Truvari was equipped with parameters “‐pick ac ‐r 2000 ‐C 5000 ‐passonly”, which represents if the breakpoint distance was smaller than 2000 bp and the allele sequence similarity was larger than 0.7, and the allele count of SV zygosity was the same, the detected SV was marked as correct. When it comes to translocations, the breakends of both SVs were focused on. If the two breakends of ground truth SV and detected SV were within 1000 bp on the same chromosome, the detected SV was marked as correct. After screening all detected SVs and ground truth SVs, the precision, recall, and F1 score of SV detection were defined as:

(18) $$Pre\_Precision=\frac{TP_{call}}{TP_{call}+FP}$$

(19) $$Pre\_Recall=\frac{TP_{base}}{TP_{base}+FN}$$

(20) $$Pre\_F1=\frac{2*Pre\_Precision*Pre\_Recall}{Pre\_Precision+Pre\_Recall}$$

(21) $$GT\_Precision=\frac{TP_{gt}}{TP_{call}+FP}$$

(22) $$GT\_Recall=\frac{TP_{gt}}{TP_{base}+FN}$$

(23) $$GT\_F1=\frac{2*GT\_Precision*GT\_Recall}{GT\_Precision+GT\_Recall}$$

where *TP_call_* and *FP* represent the number of SVs that are correct or incorrect present in the detected call sets, respectively. *TP_base_* and *FN* represent the number of SVs that are detected or undetected in the ground truth call sets, respectively. *TP_gt_* represents the number of SVs that are both detected and correctly genotyped.

## Funding

This work was supported by the National Natural Science Foundation of China (Grant Nos. 62472120, 62331012), the National Key Research and Development Program of China (Grant No. number: 2024YFC3406303) and the Key Research and Development Program of Heilongjiang Province (Grant No. 2022ZX02C20).

## Conflicts of Interest

The authors declare no conflicts of interest.

## Supporting information




**Supporting File 1**: advs74351‐sup‐0001‐SuppMat.docx.

**Supporting File 2**: advs74351‐sup‐0002‐Tables.xlsx.

## Data Availability

cuteHap was implemented in Python and can be easily installed via Bioconda and PyPI. Its source code is available at https://github.com/Meltpinkg/cuteHap under MIT open source license, and is openly available in Zenodo at https://zenodo.org/records/18346494 [46]. The simulation data were generated using our in‐house scripts, and the related variant files and scripts are available at https://github.com/Meltpinkg/Simulation‐datasets‐for‐cuteHap. The GRCh38 human reference genome is available at http://ftp.1000genomes.ebi.ac.uk/vol1/ftp/technical/reference/GRCh38_reference_genome/GRCh38_full_analysis_set_plus_decoy_hla.fa. The PacBio HiFi alignment data of the HG002, HG003 and HG004 individuals are available at https://downloads.pacbcloud.com/public/revio/2022Q4/. The ONT alignment data for the HG002 individual are available at s3://ont‐open‐data/giab_lsk114_2022.12/. The GIAB Challenging Medically Relevant Gene Benchmark (CMRG) v1.00 is available at https://ftp.ncbi.nlm.nih.gov/giab/ftp/release/AshkenazimTrio/HG002_NA24385_son/CMRG_v1.00/GRCh38/StructuralVariant/. The T2T draft benchmark set is available at https://ftp‐trace.ncbi.nlm.nih.gov/ReferenceSamples/giab/data/AshkenazimTrio/analysis/NIST_HG002_DraftBenchmark_defrabbV0.019‐20241113/. The genome stratification files are available at https://ftp‐trace.ncbi.nlm.nih.gov/ReferenceSamples/giab/release/genome‐stratifications/v3.3/. The alignment data and call sets of the Platinum Pedigree Trio are available at s3://platinum‐pedigree‐data/. The PacBio HiFi alignment of the HG00096, HG01505 are available at https://ftp.1000genomes.ebi.ac.uk/vol1/ftp/data_collections/HGSVC3/working/20220831_JAX_HiFi/. The variant data is available at https://ftp.1000genomes.ebi.ac.uk/vol1/ftp/data_collections/HGSVC3/release/Variant_Calls/1.0/GRCh38/. The PacBio HiFi and ONT alignment data of the HG008 are available at https://ftp‐trace.ncbi.nlm.nih.gov/ReferenceSamples/giab/data_somatic/HG008/Liss_lab/. The PacBio HiFi alignment data of COLO829 is available at https://downloads.pacbcloud.com/public/revio/2023Q2/COLO829/COLO829/analysis/. The ONT alignment data of COLO829 is available at https://www.ncbi.nlm.nih.gov/sra/SRX23913863. The data that support the findings of this study are openly available in Zenodo at https://doi.org/10.5281/zenodo.18346561 (repository name: VCF callsets generated by cuteHap v1.0.3, doi: 10.5281/zenodo.18346561, reference number 18346561).
