# Supplementary material for: cuteHap: Haplotype‐Aware Structural Variant Detection in Phased Long‐Read Sequencing Data
Source: Adv Sci (Weinh). 2026 Feb 12;13(22):e19314. doi: 10.1002/advs.202519314 (PMC13088286; doi:10.1002/advs.202519314)
Supplement: Supplementary file 1 — Supporting File 1: advs74351‐sup‐0001‐SuppMat.docx. [file ADVS-13-e19314-s001.docx]

**cuteHap: Haplotype-Aware Structural Variant Detection in Phased Long-Read Sequencing Data**

**Supporting information**

Shuqi Cao ^1,2^, Yadong Liu^1,2,3^, Miao Cui^1,2^, Runtian Gao^4^, Weimin Guo^1,2^, Guohua Wang^1,2^, Yadong Wang^1,2,3,*^, Tao Jiang^1,2,3 *^

**Context**

[Fig. S1. Examples on the determination of window quality in cuteHap. 3](#_Toc220074464)

[Fig. S2. Schematic illustration of two strategies in cuteHap. 4](#_Toc220074465)

[Fig. S3. F1 score of various tools under different sequencing technologies on HG002. 5](#_Toc220074466)

[Fig. S4. F1 score of various tools on different SV types on HG002 (allele-count mode). 6](#_Toc220074467)

[Fig. S5. F1 score of various tools on different SV types on HG002 (single mode). 7](#_Toc220074468)

[Fig. S6. tMCR of various tools on different trios. 8](#_Toc220074469)

[Fig. S7. An example snapshot of dual-heterozygous insertions in the Ashkenazim Trio. 9](#_Toc220074470)

[Fig. S8. The F1 score of cuteHap using different phasing tools on Platinum Pedigree Trio samples. 10](#_Toc220074471)

[Fig. S9. The F1 score of SNV calling under different sequencing coverages on simulation datasets. 11](#_Toc220074472)

[Fig. S10. The elapsed time and memory footprints of cuteHap. 12](#_Toc220074473)

[Supplementary Methods 13](#_Toc220074474)

[Supplementary Commands 17](#_Toc220074475)

**Fig. S1. Examples on the determination of window quality in cuteHap.**

These four representative IGV screenshots exemplify distinct phasing patterns that determine window quality classification in cuteHap. **a)** all reads are evenly phased into paternal and maternal haplotype groups; **b)** most reads evenly phased into paternal and maternal haplotype groups and minimal reads remain unphased; consequently, these regions in a) and b) are categorized as high-quality windows. **c)** the majority of reads are phased to a single haplotype, leaving few for the other haplotype or remaining unphased; **d)** most reads cannot be assigned to either haplotype; The phasing profiles in c) and d) fail to accurately reconstruct the underlying sequencing data, so they are classified as low-quality windows.

**Fig. S2. Schematic illustration of two strategies in cuteHap.**

This illustration provides a comprehensive overview of the core algorithms in cuteHap, which consists of two main modules: haplotype-based self-adaptive allele clustering in high-quality windows, and cluster credibility-prioritized beam search algorithm for allele classification in low-quality windows. 1) Haplotype-based self-adaptive allele clustering: cuteHap first groups all supporting signatures by their haplotype tags, forming three haplotype-specific pools. A self‑adaptive clustering strategy is then applied within each pool to identify candidate allele clusters. Subsequently, an interquartile range (IQR)-based allele identification is performed to achieve alleles of various haplotypes. Finally, alleles from different haplotypes are combined and genotyped (as homozygous, heterozygous, or mosaic) according to the haplotype existence. 2) Cluster credibility-prioritized beam search: when the haplotype tags are not reliable enough, cuteHap aggregates all SV signatures within the window. Two scanning lines traverse the discretized genomic coordinates to enumerate candidate clusters. cuteHap evaluates the credibility score for each candidate cluster. Then, cuteHap applies beam search iteratively to identify a valid cluster set that maximizes both capacity and overall credibility as the final set of detected SVs.

**Fig. S3. F1 score of various tools under different sequencing technologies on HG002.**

The ground truth comes from the T2T draft, and the benchmarks are performed by Truvari in “Single” mode. **a**. The F1 score of different tools on HiFi datasets. **b**. The F1 score of different tools on ONT datasets. **c**. The F1 score of different tools under different sequencing coverages on HiFi datasets. **d**. The F1 score of different tools under different sequencing coverages on ONT datasets.

**Fig. S4. F1 score of various tools on different SV types on HG002 (allele-count mode).**

The ground truth comes from the T2T draft, and the benchmarks are performed by Truvari in “Allele-count” mode. **a**. The F1 score of different tools on insertions on HiFi datasets. **b**. The F1 score of different tools on insertions on ONT datasets. **c**. The F1 score of different tools on deletions on HiFi datasets. **d**. The F1 score of different tools on deletions on ONT datasets.

**Fig. S5. F1 score of various tools on different SV types on HG002 (single mode).**

The ground truth comes from the T2T draft, and the benchmarks are performed by Truvari in “Single” mode. **a**. The F1 score of different tools on insertions on HiFi datasets. **b**. The F1 score of different tools on insertions on ONT datasets. **c**. The F1 score of different tools on deletions on HiFi datasets. **d**. The F1 score of different tools on deletions on ONT datasets.

**Fig. S6. tMCR of various tools on different trios.**

tMCR, defined as the harmonic mean of true discovery rate and discovery performance, enables the simultaneously consideration of both the false discovery rate of violations, and the overall discovery capacity within the trio. **a.** The tMCR of different tools on Ashkenazim Trio, which includes HG002 (child), HG003 (father), and HG004 (mother). **b.** The tMCR of different tools on Platinum Pedigree Trio, which includes NA12881 (child), NA12877 (father), and NA12878 (mother).

**Fig. S7. An example snapshot of dual-heterozygous insertions in the Ashkenazim Trio.**

The illustrated region on HG002 containing two heterozygous insertions around chromosome 1: 236,097,300, inherited from the paternal and maternal parents, respectively. The offspring sample, HG002, carries two heterozygous insertions with SV length of 5,187bp and 4,030bp according to the HG002 T2T draft. The father sample, HG003, carries the insertion allele of 4,030bp, while the mother sample, HG004, carries the insertion allele of 5,187bp. Due to their large size, these insertions pose challenges for alignment and signature extraction. cuteHap effectively distinguished insertion signatures and clipped reads at breakpoints, improving recognition accuracy. Notably, only cuteHap and SVision-pro identified both insertions in HG002, and only cuteHap detected the 5,187 bp insertion in HG004, accurately reflecting Mendelian inheritance patterns.

**Fig. S8. The F1 score of cuteHap using different phasing tools on Platinum Pedigree Trio samples.**

The F1 score of cuteHap using WhatsHap and LongPhase input on PacBio HiFi data of NA12877 and NA12878.

**Fig. S9. The F1 score of SNV calling under different sequencing coverages on simulation datasets.**

The SNV calling F1 score of Clair3 under different sequencing coverages on simulation datasets.

**Fig. S10. The elapsed time and memory footprints of cuteHap.**

**a.** The elapsed time and memory footprints of cuteHap across different sequencing coverages on PacBio HiFi HG002 datasets. **b.** The elapsed time and memory footprints of cuteHap across different sequencing coverages on ONT HG002 datasets. **c.** The elapsed time and memory footprints of cuteHap across different genome sizes on PacBio HiFi datasets. **d.** The elapsed time and memory footprints of cuteHap across different phased blocks on PacBio HiFi datasets.

**Supplementary Methods**

The pseudo-code of the haplotype-aware self-adaptive clustering algorithm is described in Algorithm S1.

| **Algorithm S1. The haplotype-aware self-adaptive clustering algorithm** | |
| --- | --- |
| Input: Signatures ($POS$, $LENGTH$, $HP$), min_support, thres, thres_dist | |
| Output: SVList | |
| 1 | function adaptive_cluster(SigsList, min_support=5, thres=0.9, thres_dist=1000): |
| 2 | Lengthclusters = [SigsList[0].LENGTH] |
| 3 | Clusters = [] |
| 4 | cur_bias = SigsList[0].LENGTH * threshold |
| 5 | for sig in SigsList[1:]: |
| 6 | if sig.LENGTH – cur_bias > thres_dist: |
| 7 | Q1=percentile(lengths, 25) |
| 8 | Q3=percentile(lengths, 75) |
| 9 | IQR = Q3 – Q1 |
| 10 | Clusters.append([Q1 – 1.5 * IQR, mean(lengths), Q3 + 1.5 * IQR]) |
| 11 | Lengthclusters.append([]) |
| 12 | endif |
| 13 | Lengthclusters.append(sig.LENGTH) |
| 14 | cur_bias = sig.LENGTH * threshold |
| 15 | end for |
| 16 | return Clusters |
| 17 | SigsGroup = { 0: list(),1: list(), 2: list() } |
| 18 | Results = list() |
| 19 | for sig in Signatures: # Group signatures by phasing categories |
| 20 | SigsGroup[sig.HP].append(sig) |
| 21 | end for |
| 22 | SigsGroup.sorted( key: sorted(value , key=lambda x: x.LENGTH) ) |
| 23 | HP_res = list() |
| 24 | for _hp in SigsGroup: |
| 25 | HP_res.extend(adaptive_cluster(SigsGroup[_hp], support, thres, thres_­dist)) |
| 26 | end for |
| 27 | for i in range(len(HP_res)): |
| 28 | for j in range(i+1, len(HP_res)): |
| 29 | # check whether i and j can be combined |
| 30 | if min(HP_res[i][1], HP_res[j][1]) / max(HP_res[i][1], HP_res[j][1]) > 0.9  and max(HP_res[i][0], HP_res[j][0]) < min(HP_res[i][2], HP_res[j][2]): |
| 31 | Results.append(merge(HP_res[i], HP_res[j])) |
| 32 | end if |
| 33 | end for |
| 34 | if HP_res[i] not combined: |
| 35 | Results.append(HP_res[i]) |
| 36 | end if |
| 37 | end for |
| 38 | return Results |

The pseudo-code of the cluster credibility-prioritized beam search algorithm is described in Algorithm S2.

| **Algorithm S2. The cluster credibility-prioritized beam search algorithm** | |
| --- | --- |
| Input: Candidates, SigsPool | |
| Output: AnswerClusters | |
| 1 | function match_intervals(clusters): |
| 2 | scores = dict() |
| 3 | for cluster in clusters: |
| 4 | scores[cluster] = list() |
| 5 | end for |
| 6 | for i in range(len(SigsPool)): |
| 7 | for j in range(len(clusters)): |
| 8 | end_pos = SigPool[i].pos + SigPool[i].length |
| 9 | length = clusters[j].length |
| 10 | k = SigPool.pos_set.lower_bound(end_pos - length) |
| 11 | new_match_score = sim_score(cluster[j], SigsPool[k: i+1]) |
| 12 | dp[i][j] = max(dp[i-1][j], dp[i][j-1], dp[k-1][j-1] + new_match_score) |
| 13 | # initial value of dp is 0 for all subscripts |
| 14 | # match choice of each dp needs to be recorded |
| 15 | end for |
| 16 | end for |
| 17 | scheme = match_scheme(dp[len(hp)-1][len(clusters)-1]) |
| 18 | for cluster in clusters: |
| 19 | if cluster in scheme: |
| 20 | scores[cluster].add(scheme[cluster].score) |
| 21 | end if |
| 22 | end for |
| 23 | final_score = 1.0 |
| 24 | for cluster in clusters: |
| 25 | if len(scores[cluster]) < 2: |
| 26 | return 0 |
| 27 | p, n = 1.0, 1.0 |
| 28 | for score in scores[cluster]: |
| 29 | p = p * score |
| 30 | n = n * (1.0 - score) |
| 31 | end for |
| 32 | final_score = final_score * (p / (p + n)) |
| 33 | end for |
| 34 | return final_score |
| 35 | k = 0 |
| 36 | Ans[0] = [set()] |
| 37 | while len(Ans[k]) > 0: |
| 38 | k = k + 1 |
| 39 | FullAnsSet = set() |
| 40 | for ans in Ans[k-1]: |
| 41 | AnsSet = set() |
| 42 | for cluster in Candidates: |
| 43 | if cluster in ans: |
| 44 | continue |
| 45 | end if |
| 46 | new_ans = set(ans + cluster) |
| 47 | if new_ans valid: |
| 48 | AnsSet.add(new_ans) |
| 49 | if len(AnsSet) >= beam_size / 2: |
| 50 | break |
| 51 | end if |
| 52 | end if |
| 53 | end for |
| 54 | FullAnsSet.extend(AnsSet) |
| 55 | end for |
| 56 | Ans[k] = sorted(FullAnsSet, key=lambda x:match_intervals(x), reverse=True)[:beam_size] |
| 57 | end while |
| 58 | return Ans[k-1][0] |

**Supplementary Commands**

The complete commands of data generation, variant calling and benchmarking implementation in this study are as below:

**1. Implementation of simulation**

1.1 Add SNPs to the reference genome (only chromosome 1 and 2 in this paper):

> VISOR HACk -b snps.h1.bed snps.h2.bed -g GRCh38.fa -o GRCh38withSNP

1.2 Add SVs to the modified haplotype1 and haplotype 2 respectively:

> VISOR HACk -b svs.h1.bed -g GRCh38withSNP/h1.fa -o GRCh38withSVH1

> VISOR HACk -b svs.h2.bed -g GRCh38withSNP/h2.fa -o GRCh38withSVH2

> samtools faidx GRCh38withSVH1/h1.fa chr1 chr2 > GRCh38withSVH1/chr1_2.fa

> samtools faidx GRCh38withSVH2/h1.fa chr1 chr2 > GRCh38withSVH2/chr1_2.fa

1.3 Simulate PacBio HiFi sequencing for the modified haplotypes respectively (haplotype1 for example):

> pbsim --strategy wgs --genome chr1_2.fa --depth 15 --method qshmm --qshmm ~/pbsim3/data/QSHMM-RSII.model --length-mean 15000 --accuracy-mean 0.99 --pass-num 5

> samtools merge -@ 8 sd_0001_2.bam sd_0001.sam sd_0002.sam -O BAM

> ccs sd_0001_2.bam sd_0001_2.ccs.fastq.gz

> minimap2 GRCh38.fa sd_0001_2.ccs.fastq.gz -a -o sim_h1.sam -t 8 -x map-pb

> samtools merge -u - sim_h1.sam sim_h2.sam | samtools sort -@ 8 -o sim_sorted.bam

> samtools index sim_sorted.bam

**2. Implementation of reads phasing**

2.1 SNP calling

> run_clair3.sh --bam_fn=${bam_file} --ref_fn=${ref_file} --threads=16 --platform=hifi --model_path=~/clair3/bin/models/hifi --output=${out_path}

> run_clair3.sh --bam_fn=${bam_file} --ref_fn=${ref_file} --threads=16 --platform=ont --model_path=~/clair3/bin/models/r941_prom_sup_g5014 --output=${out_path}

2.2 reads phasing with LongPhase

> longphase phase -s ${out_path}/merge_output.vcf.gz -b ${bam_file} -r ${ref_file} -t 16 -o longphase --pb

> longphase haplotag -r ${ref_file} -s longphase.vcf -b ${bam_file} -t 16 -o ${out_path}/longphase.tag

2.3 reads phasing with WhatsHap

> whatshap phase ${out_path}/merge_output.vcf.gz ${bam_file} --reference=${ref_file} --distrust-genotypes --ignore-read-groups -o phased.vcf.gz

> bcftools index phased.vcf.gz

> whatshap haplotag phased.vcf.gz ${bam_file} --reference=${ref_file} --ignore-read-groups -o /tag.bam --output-haplotag-list=phasedinfo.tsv --output-threads=16

**3. Implementation of variant calling**

3.1 Implement alignment-based methods

> cuteSV ${bam_file} ${ref_file} cutesv.vcf ./ --genotype -s $support_num -l 50

“--max_cluster_bias_INS 1000 --diff_ratio_merging_INS 0.9 --max_cluster_bias_DEL 1000 --diff_ratio_merging_DEL 0.5” are applied to PacBio HiFi data, and “--max_cluster_bias_INS 100 --diff_ratio_merging_INS 0.3 --max_cluster_bias_DEL 100 --diff_ratio_merging_DEL 0.3” are applied to ONT data

> sniffles --input ${bam_file} --vcf sniffles2.vcf --reference ${ref_file} -t 8

> debreak --bam ${bam_file} --outpath debreak -t 8 --min_support $support_num -r ${ref_file} --min_size 50 --poa --rescue_large_ins

> SVision-pro --detect_mode germline --target_path ${bam_file} --genome_path ${ref_file} --model_path ~/SVision-pro/src/pre_process/model_liteunet_256_8_16_32_32_32.pth --out_path SVisionpro_out --process_num 8 --min_supp $support_num

> svim alignment svim ${bam_file} ${ref_file} --min_sv_size 50

> awk -v s="$support_num" '/^#/{print; next} match($0,/SUPPORT=([0-9]+)/,a) && a[1]>=s' svim/variants.vcf > svim.vcf

> pbsv discover ${bam_file} sample.svsig.gz --hifi

> pbsv call ${ref_file} sample.svsig.gz pbsv.vcf --hifi --min-sv-length 50

3.2 Implement ensemble-based methods

> jasmine file_list=sample.txt out_file=jasmine_merge.vcf min_support=2 threads=8 --output_genotypes

> bcftools sort jasmine_merge.vcf -o jasmine_sort.vcf

> cuteFC ${bam_file} ${ref_file} cutefc.vcf ./ -Ivcf jasmine_sort.vcf -l 50

“--max_cluster_bias_INS 1000 --diff_ratio_merging_INS 0.9 --max_cluster_bias_DEL 1000 --diff_ratio_merging_DEL 0.5” are applied to PacBio HiFi data, and “--max_cluster_bias_INS 1000 --diff_ratio_merging_INS 0.5 --max_cluster_bias_DEL 1000 --diff_ratio_merging_DEL 0.5” are applied to ONT data

> sniffles --input ${bam_file} --vcf sniffles2.fc.vcf --reference ${ref_file} -t 8 --genotype-vcf jasmine_sort.vcf

3.3 Implement phase-based methods

> cuteHap ${bam_file} ${ref_file} cutehap.vcf ./ -s $support_num

*Note: “-q 0” is additionally added for simulated data.*

> duet ${bam_file} ${ref_file} duet -t 8 -r $support_num

3.4 Implement mosaic detection methods

> sniffles --input ${bam_file} --vcf sniffles2.mosaic.vcf --reference ${ref_file} -t 8 --mosaic

> cuteHap ${bam_file} ${ref_file} cutehap.mosaic.vcf ./ -s 2 --mosaic --large_inv

**4. Implementation of spike-in generation**

> bash down_hp1.sh ${bam_file} ${depth} ${downsample_file}

> samtools merge -u - ${downsample_file} ${NA12878.bam} | samtools sort -@ 8 -o merged_${depth}.bam

**5. Implementation of benchmarking**

> truvari bench -b GRCh38_HG2-T2TQ100-V1.1_stvar.vcf.gz -c ${vcf} --includebed GRCh38_HG2-T2TQ100-V1.1_stvar.benchmark.bed -o cmp --pick ac --passonly -r 2000 -C 5000 -f GRCh38.fa

> truvari refine --use-original-vcfs --align mafft -f GRCh38.fa -r cmp/candidate.refine.bed cmp -t 16 --mafft-params "--auto --thread 16"

> truvari bench -b GRCh38_HG2-T2TQ100-V1.1_stvar.vcf.gz -c ${vcf} --includebed GRCh38_HG2-T2TQ100-V1.1_stvar.benchmark.bed -o cmp -p 0 -r 1000 --passonly
